# Can pelvic lymphadenectomy be omitted in patients with stage IA2, IB1, and IIA1 squamous cell cervical cancer?

**DOI:** 10.1186/s40064-016-2927-5

**Published:** 2016-08-05

**Authors:** Yaxian Wang, Tingting Yao, Jin Yu, Jing Li, Qionghua Chen, Zhongqiu Lin

**Affiliations:** 1 Department of Gynecological Oncology, Sun Yat-sen Memorial Hospital, Sun Yat-sen University, 107 Yan Jiang West Road, Guangzhou, 510120 People’s Republic of China; 2 Xiamen Cancer Center, Department of Obstetrics and Gynecology, The First Affiliated Hospital of Xiamen University, Xiamen, 361003 People’s Republic of China

**Keywords:** Early stage cervical cancer, Squamous cell carcinoma antigen, Pelvic lymphadenectomy, Lymph node metastasis, Stromal invasion, Lymphovascular invasion

## Abstract

**Purpose:**

This study aimed to identify the surgical-pathologic risk factors of lymph node metastasis (LNM) in patients with early stage squamous cell cervical cancer and to evaluate the potential efficacy of omitting pelvic lymphadenectomy.

**Methods:**

A total of 276 patients with stage IA2, IB1, and IIA1 squamous cell cervical cancer receiving primary radical hysterectomy with pelvic lymphadenectomy were included in this study.

**Results:**

The incidences of LNM in patients with stage IA2, IB1, and IIA1 squamous cell cervical cancer were 0 % (0/8), 17.4 % (36/207), and 29.5 % (18/61), respectively. The most common location of LNM was the obturator lymph node. Human papilloma virus 16 subtype was the most common infection in early stage squamous cell cervical cancer. Univariate analysis revealed that squamous cell carcinoma antigen (SCCAg) greater than 1.5 μg/L (p < 0.001), tumor size greater than 2 cm (p < 0.001), tumor size greater than 3 cm (p < 0.001), depth of stromal invasion (p < 0.001) and lymphovascular invasion (p < 0.001) were associated with LNM. Logistic regression analysis revealed that depth of stromal invasion {model 1 [p = 0.006; odds ratio (OR) 2.161; 95 % confidence interval (CI) 1.251–3.734], model 2 [p = 0.002; OR 2.344; 95 % CI 1.337–3.989]}, lymphovascular invasion [model 1 (p = 0.004; OR 2.967; 95 % CI 1.411–6.237), model 2 (p = 0.004; OR 2.978; 95 % CI 1.421–6.243)], and SCCAg greater than 1.5 μg/L [model 1 (p = 0.023; OR 2.431; 95 % CI 1.129–5.235), model 2 (p = 0.024; OR 2.418; 95 % CI 1.125–5.194)] were independently associated with LNM.

**Conclusions:**

Pelvic lymphadenectomy may be omitted in patients with SCCAg lower than 1.5 μg/L, superficial stromal invasion and without lymphovascular invasion in stage IA2, IB1, IIA1 squamous cell cervical cancer.

## Background

Cervical cancer is a global public health problem and a significant cause of death among women in developing countries (Forouzanfar et al. 2011). The International Federation of Gynecology and Obstetrics (FIGO) recommends a clinical staging system for cervical cancer. Although lymph node status assessment does not change clinical stage, lymph node metastasis (LNM) is an independent factor in poor survival and relapse of cervical cancer (Pieterse et al. 2007; Ditto et al. 2013; Bai et al. 2015). Guidelines recommend radical hysterectomy with pelvic lymphadenectomy is the preferred treatment for early stage (FIGO IA2, IB1, IIA1) cervical cancer. However, pelvic lymphadenectomy is associated with potential risk of serious postoperative complication, such as nerve or vascular injury, lymphedema lymphedema in the lower limbs and pelvic lymph cysts with concomitant infection (Matsuura et al. 2006; Achouri et al. 2013; Kawamura et al. 2015).

One research suggest that the number of removed lymph nodes in pelvic lymphadenectomy pay no effect on the prognosis of patients with early stage cervical cancer with negative pelvic lymph nodes (Pieterse et al. 2007). Approximately 80 % of cervical cancer cases are squamous cell carcinomas. Non-squamous histology has been shown to be an independent prognostic factor for disease-free and overall survival compared with squamous cell carcinoma in cervical cancer with LNM (Kodama et al. 2006; Park et al. 2010). Thus, we speculate that pelvic lymphadenectomy may be avoided in patients with early cervical squamous cell cancer who are in low risk of LNM, to reduce associated morbidity of complete lymphadenectomy and long-term effects of postoperative complication, may help to improve the postoperative quality-of-life of patients.

Squamous cell carcinoma antigen (SCCAg) is the serum tumor marker most commonly used for clinical monitoring of cervical squamous cell cancer(Kim 2013). Elevated pre-treatment SCCAg levels correlate with LNM and a poor prognosis in cervical squamous cell cancer (van de Lande et al. 2009; Kawaguchi et al. 2013).

In the present study, we retrospectively reviewed the records of patients with stage IA2, IB1, and IIA1 cervical squamous cell cancer who received radical hysterectomy with pelvic lymphadenectomy. We examined the effect of clinicopathologic factors, also take preoperative serum SCCAg into account, on the risk for LNM to identify patients with low risk for whom pelvic lymphadenectomy may be omitted.

## Methods

### Patients

We retrospectively analyzed the data of patients with cervical cancer treated at the Department of Gynecological Oncology, Sun Yat-sen Memorial Hospital of Sun Yat-sen University between January 2012 and June 2015. This study included patients who met the following criteria: (1) FIGO IA2, IB1, or IIA1 cervical cancer and received radical hysterectomy with pelvic lymphadenectomy; (2) histological confirmed squamous cell carcinoma according to FIGO classification system; (3) no preoperative radiotherapy or chemotherapy. The study was performed in accordance with the Declaration of Helsinki and was approved by the ethics committee of the Sun Yat-sen Memorial Hospital of Sun Yat-sen University. All patients provided written consent for storage of their information in the hospital database and for use of this information in our research.

### Surgical procedures

Radical hysterectomy included complete removal of the uterus, ovaries, Fallopian tubes, cervix, upper vagina and parametrium. A systematic lymphadenectomy consisted of dissections of lymph nodes in the common iliac, external iliac, internal iliac, obturator and deep inguinal areas, at both sides. The cranial, caudal, ventral, dorsal, lateral and medial boundaries of the lymphadenectomy were 3 cm above the bifurcation of the internal and external iliac arteries, the level of deep iliac circumflex vein, the level of peritoneum, the level of the obturator nerve, the inside of the psoas major muscle and the lateral border of ureter, respectively. Para-aortic lymphadenectomy was performed only in case of gross metastasis to the common iliac nodes or para-aortic nodes was suspected.

### SCCAg assay

Serum samples were taken routinely at the patient’s initial visit before surgery for SCCAg analysis. Serum SCCAg levels were analyzed using an automated chemiluminescence immunoassay (CLIA) system (i2000, Abbott, USA) according to the manufacturer’s instructions. The recommended cutoff level of 1.5 ng/ml was applied. Ten patients had no serum SCCAg data.

### Sample collection and HPV testing

The gynecologist rotated the disposable cervical cell collection brush clockwise 3–5 times at the cervical junction to collect exfoliated cervical cells. The cervical brush was removed, stored in preservation solution, and sent to laboratory to test immediately. HPV genotypes were detected using nucleic acid molecule flow-through PCR and membrane hybridization using gene chip technology with the 21HPV Genoarray Diagnostic Kit (Hybribio Inc, Guangzhou, China). HPV nucleic acid was extracted, and gene amplification and molecular hybridization were performed, according to the manufacturer’s instructions. There were 21 HPV subtypes tested simultaneously in a membrane including 13 types of high-risk HPVs (HPV-16, 18, 31, 33, 35, 39, 45, 51, 52, 56, 58, 59, and 68), 5 low-risk HPVs (HPV-6, 11, 42, 43,and 44) and 3 HPVs that are common in Chinese people (HPV-53, 66, and CP8304). Twenty-four patients had no HPV testing data.

### Statistical analysis

The relationship between clinical and pathological factors and LNM was examined by univariate analysis using the χ^2^ and Fisher’s exact probability tests. The independent effects of clinical and pathological factors on LNM were then determined by logistic regression analysis, in which factors that were statistically significant in univariate analysis. A *p* value <0.05 was considered significant for all analyses. SPSS software (version 16.0; IBM Corporation, Armonk, NY, USA) was used for statistical analysis.

## Results

### Clinicopathologic characteristics of patients

A total of 276 patients treated for squamous cell cervical cancer between January 2012 and June 2015 met criteria for inclusion in the present study. Table 1 summarizes the clinicopatholgic characteristics of the 276 patients who had early stage cervical squamous cell cancer with a median age of 49 years (range 22–80 years). One hundred and fifty-eight patients (57.2 %) were premenopausal and 118 patients (42.8 %) were postmenopausal. Staging indicated that 8 patients (2.9 %) had FIGO IA2, 207 patients (75.0 %) had FIGO IB1, and 61 patients (22.1 %) had FIGO IIA1. Two hundred and thirty-two patients (92 %) had positive HPV infection and 68 patients (25.6 %) with SCCAg greater than 1.5 μg/L. Analysis of histological grade indicated that 98.5 % patients (272/276) had moderately to poorly differentiated cancer. Seventy-nine patients (28.6 %) had deep-full thickness stromal invasion, and 129 patients (46.7 %) with lymphovascular invasion.

**Table 1:** Clinicopathological characteristics of patient with stage IA2/IB1/IIA1 cervical squamous cell cancer (n = 276) in relation to the pelvic lymph node status

Characteristics	Total	Positive lymph node metastasis n (%)	Negative lymph node metastasis n (%)	p-value
		54	222	
Age, years				
<40	45	8 (14.8)	37 (16.7)	0.947
≥40	231	46 (85.2)	185 (83.3)	
Menopausal status				
Premenopausal	158	26 (48.1)	132 (59.5)	0.321
Postmenopausal	118	28 (51.9)	90 (40.5)	
FIGO stage				
IA2/IB1	215	36 (66.7)	179 (80.7)	0.085
IIA1	61	18 (33.3)	43 (19.4)	
HPV infection status				
Negative	20	1 (2.0)	19 (9.5)	0.180
Positive	232	50 (98.0)	182 (90.5)	
SCCAg (μg/L)				
≤1.5	198	24 (46.1)	174 (81.3)	<0.001
>1.5	68	28 (53.9)	40 (18.7)	
Histological grade				
Well differentiated	4	0 (0)	4 (1.8)	0.733
Moderately differentiated	163	34 (63.0)	129 (58.1)	
Poorly differentiated	109	20 (37.0)	89 (40.1)	
Tumor size (cm)				
≤2	124	8 (14.8)	116 (52.3)	<0.001
>2	152	46 (85.2)	106 (47.7)	
Tumor size(cm)				
≤3	225	32 (59.3)	193 (86.9)	<0.001
>3	51	22 (40.7)	29 (13.1)	
Depth of stromal invasion				
Superficial	129	6 (11.1)	122 (55.0)	<0.001
Middle	69	16 (29.6)	53 (23.9)	
Deep-full	79	32 (59.3)	47 (21.1)	
Lymphovascular invasion				
Negative	147	14 (25.9)	133 (59.9)	<0.001
Positive	129	40 (74.1)	89 (40.1)	

### HPV infection

A total of 252 patients had HPV testing data. Two hundred and thirty-two patients (92.1 %) had positive HPV infection. There were 12 HPV subtypes (HPV-16, 18, 31, 33, 39, 51, 52, 56, 58, 66 and CP8304) as detected. Single HPV subtype infections accounted for 81.0 % (188/232) of the total number of infections, and multiple-subtype infections accounted for 19.0 % (44/232), including 14.2 % (33/232) of patients with a double infection, 4.7 % (11/232) of patients with a triple or more infection. The HPV16 subtype was the most common infection (n = 190, 81.9 %), followed by HPV58 (n = 19, 8.3 %), HPV52 (n = 18, 7.9 %), HPV18 (n = 16, 7.1 %), HPV 39 (n = 11, 4.7 %).

### Lymph node metastasis

**Fig. 1:**
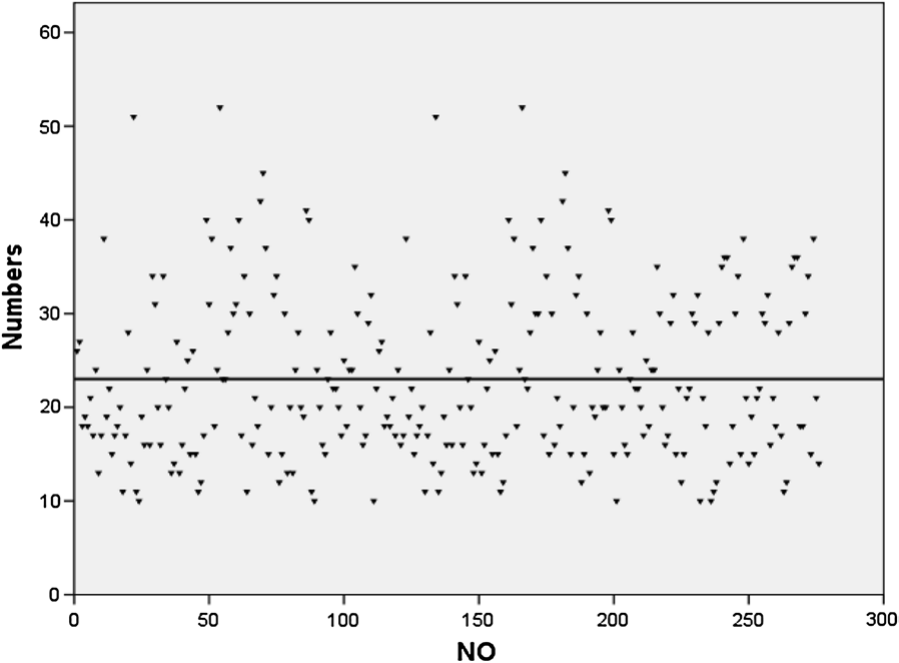
Number of removed lymph nodes per case: 1–276 women. X-axis: disease groups. Y-axis: number of removed lymph nodes. *Bar* mean

The patterns of lymph node metastasis are summarized in Table 2. The median number of dissected lymph nodes was 23 (range 10–52) (in Fig. 1). Fifty-four patients (19.6 %) had 99 positive lymph node metastasis. The incidences of LNM in patients with stage IA2, IB1, and IIA1 were 0 % (0/8), 17.4 % (36/207), and 29.5 % (18/61), respectively. Multiple (≥2) LNM was found in 10.9 % (30/276). Bilateral LNM was found in 4.7 % (13/276). Skip LNM was found in 1.1 % (3/276). The obturator lymph node was the most common location of lymph node metastasis (n = 50, 50.5 %), followed by the internal iliac lymph node (n = 16, 16.2 %), common iliac lymph node (n = 14, 14.1 %), external iliac lymph node (n = 11, 11.1 %), parametrial lymph node (n = 4, 4.0 %), inguinal lymph node(n = 3, 3.0 %) and para-aortic lymph node(n = 1, 1.0 %).

**Table 2:** Location of pelvic lymph node metastasis in 54 patients (99 positive lymph nodes) with stage IA2/IB1/IIA1 cervical squamous cell cancer

Pelvic lymph node location	Metastasis (%)
Para-aortic	1.0 (1/99)
Common iliac	14.2 (14/99)
External iliac	11.1 (11/99)
Internal iliac	16.2 (16/99)
Obturator	50.5 (50/99)
Parametrial	4.0 (4/99)
Inguinal	3.0 (3/99)

### Univariate analysis of risk factors with LNM

Table 1 shows the results of univariate analysis of the correlation between clinicopathologic factors and LNM. Patients with SCCAg greater than 1.5 μg/L (p < 0.001), tumor size greater than 2 cm (p < 0.001), tumor size greater than 3 cm (p < 0.001), depth of stromal invasion (p < 0.001) and lymphovascular invasion (p < 0.001) were significantly associated with LNM. Age, menopausal status, FIGO stage, HPV infection status and histological grade were not associated with LNM (p > 0.05).

### Multivariate analysis of risk factors with LNM

Table 3 shows multivariate analysis of the clinicopathological factors associated with the LNM in which factors that were statistically significant in univariate analysis using 2 models. The results showed that depth of stromal invasion {model 1 [p = 0.006; odds ratio (OR) 2.161; 95 % confidence interval (CI) 1.251–3.734], model 2 [p = 0.002; OR 2.344; 95 % CI 1.337–3.989]}, lymphovascular invasion [model 1 (p = 0.004; OR 2.967; 95 % CI 1.411–6.237), model 2 (p = 0.004; OR 2.978; 95 % CI 1.421–6.243)], and SCCAg greater than 1.5 μg/L [model 1 (p = 0.023; OR 2.431; 95 % CI 1.129–5.235), model 2 (p = 0.024; OR 2.418; 95 % CI 1.125–5.194)] were significantly and independently associated with LNM. Tumor size were not significantly associated with LNM (p > 0.05).

**Table 3:** Logistic regression analysis of the clinicopathological variables associated with the pelvic lymph node status using 2 models

Variables	OR	95 % CI	p
Model 1			
Depth of stromal invasion	2.161	1.251–3.734	0.006
Lymphovascular invasion	2.967	1.411–6.237	0.004
SCCAg (>μg/L)	2.431	1.129–5.235	0.023
Tumor size (>2 cm)	2.277	0.909–5.706	0.079
Model 2			
Depth of stromal invasion	2.344	1.337–3.989	0.002
Lymphovascular invasion	2.978	1.421–6.243	0.004
SCCAg (>μg/L)	2.418	1.125–5.194	0.024
Tumor size (>3 cm)	1.761	0.797–3.890	0.162

## Discussion

In the present study, the incidences of LNM in patients with stage IA2, IB1, and IIA1 cervical squamous cell cancer were 0 % (0/8), 17.4 % (36/207), and 29.5 % (18/61), respectively. The result is similar to the previous studies that LNM in cervical cancer was found in 0–20.3 % of patients with stage IA2-IB1 and in 16–38.6 % of patients with stage IIA1 (Hongladaromp et al. 2014; Togami et al. 2014; Liu et al. 2015; Zhou et al. 2015). In patients without LNM, the number of removed lymph nodes in pelvic lymphadenectomy was not associated with 5-year disease-free survival (Suprasert et al. 2012). But there are some long-term postoperative complication of pelvic lymphadenectomy, the overall incidence of symptomatic postoperative lymphocysts was 34.5 % (Achouri et al. 2013), and the incidence of lower-limb lymphedema increased over time, 12.9 % at 1 year, 20.3 % at 5 years, and 25.4 % at 10 years (Hareyama et al. 2015), both of which significantly harmed the quality-of-life. However, compared to undergoing open surgery, pelvic lymphadenectomy had a less impact on patients undergoing laparoscopic surgery, less blood loss, a lower transfusion rate, less lymphocele infection and other complications (Xiao and Zhang 2015; Bogani et al. 2014). Nevertheless, it means more than 60 % of patients in early stage cervical cancer without LNM will derive no benefit from pelvic lymphadenectomy and that it may be safely avoided in such patients. It is important to identify factors associated with the risk of LNM to reduce unnecessary pelvic lymphadenectomy.

Results of our multivariate analysis suggested that in 276 patients with stage IA2, IB1, and IIA1 cervical squamous cell cancer who received radical hysterectomy with pelvic lymphadenectomy, SCCAg greater than 1.5 μg/L, deep-full thickness stromal invasion and lymphovascular invasion were significant and independent factors for LNM, while tumor size were not significantly associated with LNM (p > 0.05).

Several studies found that lymphovascular invasion, and deep stromal invasion were statistically independently associated with LNM in early stage cervical cancer (Bai et al. 2015; Li et al. 2015; Zhou et al. 2015). One research demonstrated that patients with cervical cancer and found that stage IIA and advanced histological grade increased the risk for LNM (Li et al. 2012). Another also studied patients with stage IA–IIB squamous cell cervical cancer and found lymphovascular invasion, parametrial invasion, and depth of stromal invasion were identified as independent clinicopathological risk factors for LNM (Liu et al. 2015). But they did not analysis the SCCAg data. It had been found that SCCAg greater than 1.65 μg/L can predict LNM more accurately in stage IB1 (76 %) than in stage IIA (53 %) (van de Lande et al. 2009). In our study, SCCAg greater than 1.5 μg/L was significant and independent factors for LNM in stage IA2, IB1, and IIA1 squamous cell cervical cancer.

One prior study by Togami investigated patients with stage IA2-IIB cervical cancer and found that parametrial involvement and primary tumor size greater than 2 cm increased the risk of LNM (Togami et al. 2014). A survey by the Japan Clinical Oncology Group found that in patients with stage IB1 with clinical tumor diameter ≤2 cm, which available by magnetic resonance imaging (MRI) or specimens by cone biopsy, had less LNM and more favorable 5-year overall survival (Kato et al. 2015).As MRI is a more accurate noninvasive modality for preoperative evaluation of tumor size compared with pelvic examination (Zhang et al. 2014), in our study, tumor size which was evaluated by pelvic examination was not an independent factor for LNM.

Sentinel lymph node (SLN) mapping and biopsy has already been used in cervical cancer. A systematic review reported that SLN biopsy has the highest detection rate when 99 mTc is used in combination with blue dye (97 %), and a sensitivity of 92 % (van de Lande et al. 2007). However, the detection of sentinel lymph nodes requires an injection of technetium or blue dye and frozen section analysis. The sensitivities of SLN in diagnosing micrometastases were 81 % and the false negative rate of intra-operative frozen section examination was up to 44 % (Slama et al. 2013), while another study from Canada reported that intra-operative frozen examination of SLN accurately predicts the status of pelvic lymph nodes. The sensitivity for the detection of macro and micrometastatic disease was 88.9 % and negative predictive value was 98.8 % (Martínez et al. 2013). Recently, Indocyanine green (ICG), has been introduced in SLN mapping. A meta-analysis found that ICG SLN mapping had higher overall and bilateral detection rates while compared with blue dyes, and there was no difference while compared with combination of blue dyes and 99mTc (Ruscito et al. 2016). A study from Italy also found that in women with early stage cervical cancer, bilateral mapping was achieved in 98.5 % for ICG and 76.3 % for Tc-99m with blue dye (Buda et al. 2016). Undergoing laparoscopic surgery, ICG SLN mapping in cervical cancer also provided high overall and bilateral detection rates (Imboden et al. 2015). However, compared to patients with uterus in situ, SLN mapping in occult cervical cancer patients has lower detection rates, these patients needed proper management (Papadia et al. 2016). A diagnostic review found that early stage cervical cancer patients (FIGO IA2, IB1, IIA1) who had bilateral negative SLN result had a residual risk of 0.08 % (1/1257) on occult metastases, recommended not to perform a full pelvic lymphadenectomy in these patients (Tax et al. 2015). Further research should be made in order to standardize this method and confirm whether SLN biopsy can be used to replace pelvic lymphadenectomy for patients with early stage cervical cancer.

The retrospective nature of this study is its major limitation. Moreover, it was a single-center study and the sample was small, so our patients should not be considered representative of the general population. However, although SCC-Ag was one preoperative clinicopathologic factor in the prediction of LNM, depth of stromal invasion and lymphovascular invasion was evaluated after conization but mainly on the basis of postoperative factors.

## Conclusions

In conclusion, depth of stromal invasion, lymphovascular invasion and SCCAg greater than 1.5 μg/L were risk factors with LNM in patients with stage IA2, IB1, and IIA1 squamous cell cervical cancer. We propose pelvic lymphadenectomy maybe ommitted in patients with superficial stromal invasion, SCCAg lower than 1.5 μg/L and without lymphovascular invasion and thereby reduce the incidence of postoperative complications. More studies are needed to confirm our findings.

## Abbreviations

LNM: lymph node metastasis
HPV: human papilloma virus
SCCAg: squamous cell carcinoma antigen
FIGO: The International Federation of Gynecology and Obstetrics
